# Efficacy of Supplementing Lemongrass Powder on Growth, Metabolism, Immune and Endo‐Parasitic Status of Lambs in the Tropics

**DOI:** 10.1002/vms3.70714

**Published:** 2025-12-02

**Authors:** Md. Aliar Rahman, Most Sabina Yasmin, Peter Wynn, Rakhi Chowdhury, Khan Md. Shaiful Islam, Mohammad Al‐Mamun

**Affiliations:** ^1^ Feed Safety and Phyto Nutrition Lab Department of Animal Nutrition Bangladesh Agricultural University Mymensingh Bangladesh; ^2^ Gulbali Institute for Agriculture Water and Environment Charles Sturt University Wagga Wagga Australia

**Keywords:** endo‐parasites status, growth velocity, immune, lambs, lemongrass, liver health

## Abstract

**Background:**

Parasitic infections are prevalent among lambs in the subtropics, adversely affecting immunity, liver health and growth performance. To efficiently combat these issues, herbs as supplements are an effective approach.

**Objectives:**

The present study aims to evaluate the efficacy of lemongrass powder on growth performance, nutrient digestibility, blood biomarkers, liver enzymes, immune and endo‐parasitic status in lambs (*Ovis aries*).

**Methods:**

Ten lambs (body weight [BW]: 16.67 ± 3.82 kg) were randomly assigned to two diets (5 lambs/diet) in a crossover design, consisting of two 35‐days feeding periods separated by a 14‐days washout interval. The basal diet had 14.90% crude protein and 10.57 mega joules of metabolizable energy/kg of dry matter (DM) and was considered the control diet (CL‐diet), and the lemongrass diet (LS‐diet) consisted of the basal diet + 0.50 g of lemongrass powder/kg of metabolic BW of a lamb. Feed, faeces and blood samples were collected during each feeding period.

**Results:**

Lambs receiving the LS‐diet presented a better propensity of DM consumption (*p* = 0.07), greater average daily gain (*p* = 0.06), while showing a better feed conversion ratio (*p* = 0.05) and higher growth velocity (*p* = 0.02) compared to the CL‐diet. In addition, lambs given the LS‐diet improved nutrient digestibility, serum high‐density lipoprotein cholesterol and albumin, while reducing serum triglycerides, low‐density lipoprotein cholesterol and globulin compared to the CL‐diet (*p* < 0.05). However, feeding the LS‐diet to lambs had no impact on serum total protein, immunoglobulin (Ig)‐M, alanine aminotransferase and gamma‐glutamyltransferase, while substantially improving serum IgG and reducing aspartate aminotransferase and alkaline phosphatase, thereby improving immunity and liver health. Lambs given the LS‐diet effectively suppressed stomach worms, *Paramphistomum* spp. and *Eimeria* spp. (*p* < 0.05).

**Conclusions:**

Lemongrass powder as a supplement with a basal diet had a positive impact on growth velocity, digestibility, serum cholesterol levels, liver health, immunity and endo‐parasitic status in lambs.

## Graphical abstract

Dietary supplementation of lemongrass powder (0.50 g per kg metabolic body weight) with a basal diet significantly enhanced lamb daily gain, growth velocity, feed efficiency and nutrient digestibility. It increased serum high‐density lipoprotein cholesterol (HDL‐C), albumin and IgG concentrations, while reducing aspartate aminotransferase (AST), alkaline phosphatase (ALP), total cholesterol, low‐density lipoprotein cholesterol (LDL‐C) and endo‐parasitic load, highlighting its potential as a natural supplement in tropical lamb production.

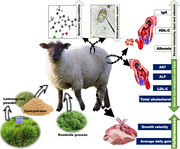

## 1 Introduction

Lamb husbandry is gaining significant attention among farmers in Asia due to its rapid return on investment. However, lambs in these regions often involve substandard practices, including poor housing, nutrition and management. In addition, extreme environmental conditions, such as severe hot and cold temperatures, alongside subclinical and clinical diseases, particularly parasitic infections, further hinder liver health, immunity, performance and profitability in lamb farming (Kļaviņa et al. 2021; Reza et al. 2021). Herbs and their extracts containing various phytochemicals have gained widespread recognition as effective supplements for reducing parasitic infection and improving liver health, immunity, nutrient digestibility and growth performance in the subtropics (Kholif et al. 2017). These phytochemical‐rich herbs, such as rosemary, spearmint, sage and lemongrass, are widely available in the subtropics. These herbs have been reported to positively influence the rumen microbiome, improve beneficial blood metabolites and enhance the immunity, liver health, performance and product quality of lambs (Allam and El‐Elaime 2020; Kholif et al. 2021).

Lemongrass (*Cymbopogon citratus*), rich in citral, limonene, tannin, β‐myrcene, saponin and flavonoid, is a perennial herb widely used as an effective feed supplement in ruminants and non‐ruminants (Rahman et al. 2022; Rahman, Redoy, Chowdhury, et al. 2024). Due to containing various phytochemicals, it exhibits antioxidant, antibacterial, anti‐inflammatory and antimicrobial properties (Wanapat et al. 2008; Rahman, Redoy, Shuvo, et al. 2024). Daily supplementation of lemongrass powder in beef cattle (100 g/head per day or 1.69 g/kg metabolic body weight [MBW] per day) improves nutrient digestibility, favourable rumen microbiome and growth performance (Wanapat et al. 2008). Furthermore, feeding daily 10 (0.60 g/kg MBW), 4 (0.23 g/kg MBW), or 5–10 (0.32–0.64 g/kg MBW) g of lemongrass powder improves the beneficial rumen microbiome, favourable blood metabolites, milk and component yield and unsaturated fatty acid contents in Damascus goats (Kholif et al. 2017), ewes (Abdullah et al. 2020) and Farafra ewes (Kholif et al. 2021), respectively. In contrast, finisher lambs receiving lemongrass powder daily (10% of the diet or 10 g/kg MBW) exhibit insignificant effects on these parameters (Bhatt et al. 2021).

Dietary supplementation of lemongrass powder at the level of 1.0% per kg of dry matter (DM) in Awassi lambs decreases the levels of serum liver enzymes and increases lymphocytes counts (Al‐Janabi et al. 2023), and 1.05 g per kg of MBW in lactating cows reduces serum liver enzymes and improves immunoglobulin (Ig) levels (Rahman, Redoy, Chowdhury, et al. 2024), nutrient digestibility, antioxidant status and milk production (Rahman, Redoy, Shuvo, et al. 2024). In contrast, supplementing lemongrass (1.26 g/kg MBW per day) has no effects on serum liver enzymes and immunity (IgG and IgM) in growing Holstein steers (Hosoda et al. 2006) and Barki goats (Khattab et al. 2017). Nonetheless, citral, a major phytochemical of lemongrass, improves the liver function by decreasing the levels of alanine and aspartate aminotransferase (AST) in the serum of rats (Li et al. 2018). Furthermore, the water extract of lemongrass has anthelmintic effect on earthworms in an in vitro study (Sherwani et al. 2013); whereas supplementing phytogenic feed with limonene reduces faecal egg count of gastrointestinal nematodes (Varga‐Visi et al. 2023). The lemongrass essential oil nano‐emulsion at a dose of 44.30 mg per kg of MBW has been reported to reduce gastrointestinal nematodes, mainly *Haemonchus contortus*, in lambs (Macedo et al. 2019).

From the findings of the above studies (Khattab et al. 2017; Kholif et al. 2021; Bhatt et al. 2021; Al‐Janabi et al. 2023), it has been demonstrated that the MBW of lambs and the administered dose of lemongrass are key factors influencing variations in performance, digestibility, serum liver enzyme activity and immune responses. However, to the best of our knowledge, no studies have specifically accounted for the MBW of lambs when supplementing with lemongrass. In addition, it is hypothesized that phytochemicals, primarily citral in lemongrass, may be available in the gut and suppress endo‐parasites or be absorbed into the bloodstream from the gut, positively altering blood metabolites, liver enzymes and immune status in lambs.

Therefore, this research was designed to assess the response of lemongrass (0.50 g/kg MBW per day) as a supplement to improve growth performance, nutrient digestibility, blood biomarkers, liver enzymes, immunity and endo‐parasitic status in lambs.

## 2 Materials and Methods

### 2.1 Experimental Design, Diet and Management

Ten non‐descript mixed sex (intact male = 6 and female = 4) lambs (*Ovis aries*) indigenous to Bangladesh, aged 1 year and weighing 16.67 ± 3.82 kg, were randomly assigned in a crossover design, including an adaptation and washout periods. Among the ten lambs, five were given a basal diet without lemongrass and considered as a control diet (CL‐diet), while the remaining five were given a lemongrass diet (LS‐diet) for 35 days (7 days adaptation and 28 days data collection periods). Following a 14 days as washout period with only a basal diet, the diets were switched for the final 35 days (7 days adaptation and 28 days data collection periods): The lambs previously on the CL‐diet were given the LS‐diet, and those on the LS‐diet were switched to the CL‐diet. The basal diet consisted of roadside grasses and concentrate ingredients (wheat bran, crushed corn, mustard oil cake and common salt), containing 14.90% crude protein (CP) and 10.57 mega joules metabolizable energy per kg of DM. The LS‐diet consisted of a basal diet supplemented with 0.50 g lemongrass powder per kg of lamb MBW. In order to ensure the appropriate dose, lemongrass was administered daily to the lambs based on their MBW. The ingredient composition of the CL‐diet, along with the nutrient compositions of both the CL‐ and LS‐diets, is presented in Table 1.

**TABLE 1:** Feed ingredients of CL‐diet and nutritive values of CL‐ and LS‐diets for lambs^a^.

Ingredients	CL‐diet (g/kg dry matter)
Roadside grass^b^	600
Wheat bran	260
Crushed corn	100
Mustard oil cake	35
Common salt (NaCl)	5

**Nutrients composition** (g/kg dry matter)	**CL‐diet**	**LS‐diet**
Organic matter	876.0	880.1
Crude protein	149.0	149.2
Crude fibre	209.0	210.3
Neutral detergent fibre	471.0	474.0
Acid detergent fibre	239.0	240.9
Ether extract	36.01	36.24
Ash	124.0	124.4
Nitrogen free extract	482.0	484.3
Metabolizable energy^c^ (mega joules)	10.57	10.61

Abbreviations: CL‐diet, Control diet; g/kg, Gram per kilogram; LS‐diet, Lemongrass diet consisting of basal diet (CL‐diet) + 0.50 g lemongrass powder/kg metabolic body weight of a lamb.

^a^ Non‐descript lambs *(O. aries*) indigenous to Bangladesh.

^b^ Roadside grass predominating *Axonopus compressus*, *Panicum repens*, *Imperata cylindrica*, *Cynodon dactylon*, *Cyperus rotundus* species.

^c^ Calculated value.

Each lamb was housed individually in a 1 × 2 m pen within a semi‐enclosed facility. Each cage was equipped with a feeder and a waterer to supply diet (roughage and concentrate mixture) and water, respectively. Each lamb was given a diet individually twice a day, with ad libitum access to clean and fresh water. The powdered lemongrass was thoroughly mixed with each lamb's concentrate mixture in the morning before the roughage supply, and complete consumption was ensured through direct observation. For the preparation of lemongrass (*C. citratus* DC. Stapf) powder, the herb was cultivated using optimal agricultural practices and harvested at 65 days (Rahman and Chowdhury 2025). Then, it was dried in a shaded area for 4–5 days at 27.0 ± 3.0°C using a pedestal fan, following the optimal wilting process. Subsequently, the dried lemongrass was ground into a fine powder using a locally available electric grinder fitted with a 1 mm sieve. Before the 15 days of adaptation, Duozol DS Vet bolus (Incepta Pharmaceuticals Ltd., Bangladesh) was administered orally at a dose of 120 mg of triclabendazole and 80 mg of levamisole per 10 kg of body weight (BW) to control gastrointestinal nematodes.

### 2.2 DM Intake, Nutrient Digestibility and Blood Collection

Daily roughage intake was calculated by subtracting the refused roughage from the amount provided, while concentrate consumption was recorded separately. Using a weighing balance, the initial and weekly BW were taken. The average daily gain (ADG) is determined by dividing the total BW gain by the number of days from initial to final BW, while the feed conversion ratio (FCR) is calculated by dividing the daily DM intake by the ADG. The growth velocity was computed by dividing the BW gain by the initial BW (Chowdhury et al. 2022). Total faeces collection was employed to determine apparent nutrient digestibility. Faeces from each lamb were collected with continuous and strict monitoring over the last 7 days of each period, stored daily in covered cans, and weighed using a balance. After thoroughly mixing the daily faeces of each lamb, one subsample (5–10 g) was immediately taken and dried in an oven at 105°C for 24 h (AOAC 2005) for DM determination, while another subsample (25–30 g) was stored at −20°C. The seven days' stored samples for each lamb were later thawed, oven‐dried at 60°C and homogenously mixed to form a composite sample. This composite was ground using a locally manufactured electric grinder equipped with a 1 mm sieve. Then, apparent nutrient digestibility was calculated as described by Rahman, Redoy, Shuvo, et al. (2024).

In addition, blood was collected from the jugular vein using a 22‐gauge needle and syringe, then transferred into a 15 mL sterile marked Falcon tube. The blood samples were centrifuged at 3000 × *g* for 15 min using a centrifuge machine (Hermle Z 306, Germany) to harvest the serum (Rahman, Redoy, Chowdhury, et al. 2024), which was then stored in Eppendorf tubes at −20°C for analysis.

### 2.3 Samples Analysis

The proximate components (DM, CP, crude fibre, ether extract and ash) of the CL‐diet, lemongrass powder and faecal samples were determined, following the methods outlined in AOAC (2005). The nitrogen‐free extract (NFE) was calculated based on the following formula: NFE (% DM) = 100 − % of (CP + ether extract + crude fibre + ash), whereas the organic matter (OM) was determined using a formula (Rahman, Redoy, Shuvo, et al. 2024). In addition, the CL‐diet and lemongrass powder were analysed to determine their neutral and acid detergent fibre contents (Goering and Van Soest 1970). Beside, serum samples were thawed, while reagents were kept in the environment until room temperature. Afterwards, standards supplied with each kit were used to calculate the correction factor of each component of serum lipid profiles, protein indices and liver enzymes. In brief, serum profiles, that is, triglycerides, total cholesterol and high‐density lipoprotein (HDL) cholesterol were determined using enzymatic colorimetric assay kits (HUMAN GmbH, Germany) following the manufacturer's instructions by a Bioanalyzer (Urit‐810; URIT Medical Electronic Group Co., Ltd., China). For triglycerides and total cholesterol determination, 1.0 mL of the respective reagent was pipetted into separate cuvettes and mixed with 10 µL of serum, followed by incubation at room temperature for 10 min. Then, the absorbance was measured at 546 nm for both triglycerides and total cholesterol separately against the reagent blank at 37°C. For HDL‐cholesterol analysis, 1.0 mL of reagent was mixed with 0.50 mL of serum sample and centrifuged at 5000 × *g* for 10 min using a centrifuge (Hermle Z 306, Germany). The clear supernatant was carefully collected, and 50 µL was added to 1.0 mL of reagent. The mixture was incubated at room temperature for 10 min, and absorbance was measured at 546 nm while maintaining 37°C. In addition, with the same Bioanalyzer and the same company kit instructions, serum total protein and albumin concentrations were determined by the Biuret and Bromocresol Green (BCG) dye‐binding method at Endpoint, respectively. Briefly, 20 µL of serum was mixed with 1.0 mL of Biuret reagent, incubated for 10 min at room temperature, and the absorbance was measured at 546 nm with the system maintained at 37°C for the quantification of serum total protein. A 10 µL of serum was combined with 1.0 mL of BCG reagent, incubated for 5 min at room temperature and the absorbance was recorded at 546 nm against a reagent blank maintained at 37°C. In addition, serum urea concentration was determined using the same Bio‐analyzer by a kinetic method. For the preparation of reagent‐1, enzyme and reagent were mixed at a 1:100 ratio. Subsequently, 10 µL of serum was added to 1.0 mL of reagent‐1 and incubated for 5 min at room temperature. Thereafter, 1.0 mL of reagent‐2 was added to the mixture, followed by a 10 min incubation, and the absorbance was measured at 578 nm while maintaining 37°C. Then, serum low‐density lipoprotein (LDL) cholesterol, globulin and urea nitrogen levels were calculated (Rahman, Redoy, Shuvo, et al. 2024). Serum immunoglobulin G (IgG) and M (IgM) were analysed using specific kits from Sigma‐Aldrich (St. Louis, MO, USA) and through enzyme‐linked immunosorbent assay (ELISA; EL‐10A; Biobase, China) according to Rahman, Redoy, Chowdhury, et al. (2024). Serum alanine aminotransferase (ALT), AST, alkaline phosphatase (ALP) and gamma‐glutamyltransferase (GGT) activities were determined using commercial assay kits (Human Company, USA) on the same Bioanalyzer by the endpoint method. For the determination of these liver enzymes in serum, the working reagents were prepared by mixing the respective kit buffer and substrate in a 4:1 ratio. For ALT and AST assays, 1.0 mL of the corresponding working reagent was dispensed into cuvettes, followed by the addition of 50 µL of serum. In case of ALP and GGT assays, 1.0 mL of the respective working reagent was similarly dispensed into cuvettes, followed by the addition of 20 and 10 µL of serum, respectively. After incubation for 1 min at room temperature, the absorbance was measured at 340 nm for ALT and AST, and at 405 nm for ALP and GGT, using an Urit‐810 Bioanalyzer, while maintaining the temperature at 37°C. The eggs per gram of faeces for stomach worms, *Paramphistomum* spp., and the oocysts per gram of faeces for *Eimeria* spp., were determined using Stoll's Ova counting technique (Thienpont et al. 1986). All of the above samples were analysed in triplicate.

### 2.4 Statistical Analysis

The data were organized and managed in Excel. The experiment was conducted using a crossover design, and the data were analysed using SPSS 22.

Yijk=μ+Pi+Lj+Dk+eijk

where *Yijk* is response variables, *μ* is overall mean of the response variables, *Pi* is number of periods (*i* = 1, 2), *Lj* is number of lambs or experimental units (*j* = 1, 2, …10), *Dk* is number of diets (*k* = 1, 2) and *eijk* is residual error term or random error.

Then, a correlation matrix among immunity (IgM and IgG), liver health (AST and ALP), endo‐parasites (stomach worms and rumen fluke), digestibility (OM and CP) and growth performance (FCR and BW gain) was drawn to assess the relationship among these parameters. Based on the value of the Pearson correlation coefficients (*r*), the relationship between the variables was determined as follows: | *r* | < 0.50 was denoted as weak correlation, 0.50 < | *r* | < 0.75 as moderate correlation, and 0.75 < | *r* | < 0.99 as strong correlation. The results of all variables except the endo‐parasites count were presented as mean values with standard deviation, whereas the value of endo‐parasites was displayed as mean with standard error. Differences of *p* ≤ 0.05 were considered statistically significant, while values with 0.05 < *p* ≤ 0.10 were considered a trend toward significance.

## 3 Results

### 3.1 Growth Performance

**FIGURE 1:**
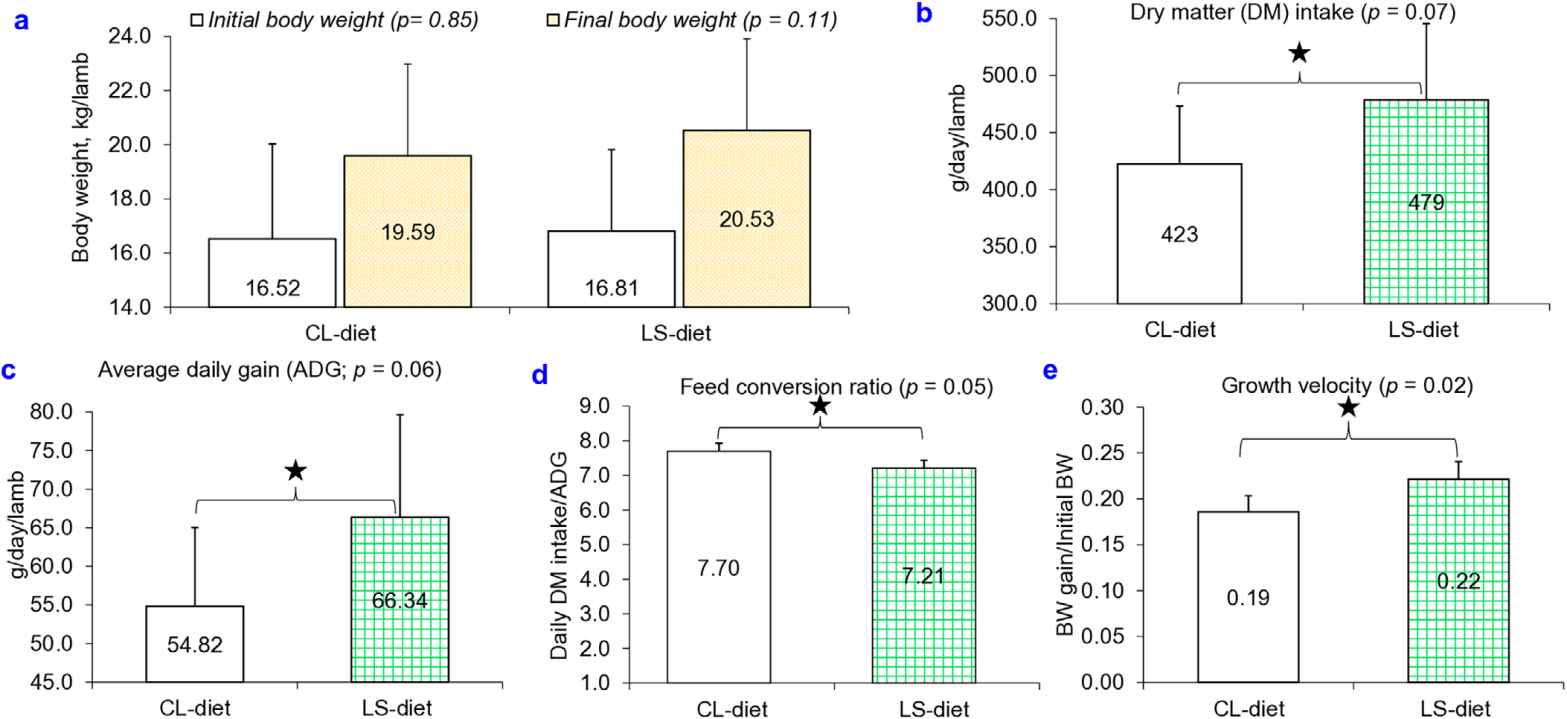
Influence of lemongrass supplementation on growth performance of lambs^†. †^Non‐descript lamb *(O. aries*) indigenous to Bangladesh. CL‐diet: Basal diet (roughage and concentrates having 14.90% crude protein and 10.57‐mega joule metabolizable energy/kg dry matter) without lemongrass supplement; LS‐diet: Basal diet + 0.50 g lemongrass powder/kg metabolic body weight of a lamb containing 14.92% crude protein and 10.61‐mega joule metabolizable energy/kg dry matter. g: Gram; kg: Kilogram; *p* ≤ 0.05 denotes statistically significant; *p ≤* 0.10 denotes a tendency to be statistically significant.

Feeding LS‐diet to lambs did not influence the final BW (*p* = 0.11; Figure 1a), while DM intake (*p* = 0.07; Figure 1b) and ADG (*p* = 0.06; Figure 1c) of lambs showed a better tendency in the LS‐diet compared to the CL‐diet. Moreover, compared to the CL‐diet, lambs given the LS‐diet showed 21.01% greater ADG. Lambs fed the LS‐diet exhibited a significantly better FCR (*p* = 0.05) compared with those on the CL‐diet (Figure 1d). Growth velocity in lambs fed the LS‐diet was 19.10% greater than that of lambs fed the CL‐diet (*p* = 0.02; Figure 1e).

### 3.2 Nutrient Digestibility

Lambs receiving the LS‐diet substantially accelerated the OM, DM, CP and ether extract digestibility by 3.80, 3.98, 4.17 and 2.48%, respectively, compared to lambs fed the CL‐diet (*p* < 0.05; Table 2). Lambs offered the LS‐diet showed a greater tendency for crude fibre digestibility compared to the CL‐diet (*p* = 0.06).

**TABLE 2:** Influence of lemongrass supplementation on apparent nutrient digestibility of lambs^a^.

Nutrient digestibility (%)	Diets^b^		SEM	*p*‐value
	CL‐diet	LS‐diet		
Lamb number (*n*)	10	10		
Dry matter	69.86 ± 2.28	72.64 ± 2.56	0.61	0.04
Organic matter	72.93 ± 1.90	75.70 ± 1.70	0.72	0.04
Crude protein	66.85 ± 1.19	69.64 ± 1.77	0.65	0.04
Crude fibre	53.89 ± 0.53	55.12 ± 0.85	0.32	0.06
Ether extract	79.84 ± 2.03	81.82 ± 0.53	0.27	0.01

*Note*: % = Percentage; *p* ≤ 0.10 denotes a tendency to be statistically significant.

Abbreviation: SEM, Standard error of mean.

^a^ Non‐descript lambs *(O. aries*) indigenous to Bangladesh.

^b^ **Diets**: CL‐diet—basal diet (roughage and concentrates having 14.90% crude protein and 10.57‐mega joule metabolizable energy/kg dry matter) without lemongrass supplement; LS‐diet—basal diet + 0.50 g lemongrass powder/kg metabolic body weight of a lamb containing 14.92% crude protein and 10.61‐mega joule metabolizable energy/kg dry matter.

### 3.3 Serum Lipid Profiles and Immune Status

Compared to the lambs fed on the CL‐diet, offering the LS‐diet to lambs decreased serum total cholesterol, triglycerides and LDL‐cholesterol concentrations by 6.42%, 11.70% and 21.23%, respectively (*p* < 0.05; Table 3). Lambs fed the LS‐diet considerably indicated a 9.46% greater HDL‐cholesterol concentration compared to lambs receiving the CL‐diet (*p* = 0.02). Serum urea nitrogen and total protein concentrations were unaffected (*p* > 0.05), while lambs that received the LS‐diet had considerably reduced serum globulin and improved serum albumin and albumin:globulin concentrations compared to lambs fed the CL‐diet. Lambs receiving the LS‐diet showed the tendency of elevated serum IgM concentrations compared to the CL‐diet (*p* = 0.07). Moreover, a significant increase in serum IgG levels was observed in the LS‐diet relative to the CL‐diet (*p* = 0.01).

**TABLE 3:** Influence of lemongrass supplementation on serum lipid profile and immune status of lambs^a^.

Variables	Diets^b^		SEM	*p*‐value
	CL‐ diet	LS‐diet		
Lamb number (*n*)	10	10		
**Serum lipid profiles (mg/dL)**				
Total cholesterol	102.98 ± 5.24	96.37 ± 3.79	1.34	0.02
Triglycerides	38.20 ± 1.93	33.73 ± 2.27	0.93	0.03
High‐density lipoprotein cholesterol	47.34 ± 7.51	51.82 ± 8.05	0.81	0.02
Low‐density lipoprotein cholesterol	48.00 ± 2.71	37.81 ± 5.57	0.60	<0.01
**Serum immune status (mg/dL)**				
Urea nitrogen	13.87 ± 0.23	13.90 ± 0.28	0.05	0.65
Total protein	6.73 ± 0.09	6.72 ± 0.12	0.03	0.83
Albumin	3.45 ± 0.05	3.56 ± 0.08	0.02	0.04
Globulin	3.28 ± 0.05	3.16 ± 0.06	0.01	0.01
Albumin:Globulin	1.05 ± 0.01	1.13 ± 0.03	0.01	0.02
Immunoglobulin‐M	172.8 ± 4.09	178.3 ± 4.14	1.53	0.07
Immunoglobulin‐G	2359 ± 37	2445 ± 46	19.05	0.01

*Note*: *p* ≤ 0.10 denotes a tendency to be statistically significant.

Abbreviations: mg/dL = Milligram per decilitre; SEM = Standard error of mean.

^a^ Non‐descript lambs *(O. aries*) indigenous to Bangladesh.

^b^ **Diets**: CL‐diet—basal diet (roughage and concentrates having 14.90% crude protein and 10.57‐mega joule metabolizable energy/kg dry matter) without lemongrass supplement; LS‐diet—Basal diet + 0.50 g lemongrass powder/kg metabolic body weight of a lamb containing 14.92% crude protein and 10.61‐mega joule metabolizable energy/kg dry matter.

### 3.4 Liver Enzymatic Activity and Endo‐Parasitic Status

Lambs fed the LS‐diet demonstrated a significant reduction in serum AST (*p* = 0.02) and ALP (*p* = 0.01) concentrations compared with those receiving the CL‐diet. However, no significant effects were observed on serum ALT (*p* = 0.13) and GGT (*p* = 0.11) levels between the two dietary groups (Table 4). In addition, the LS‐diet substantially reduced the faecal egg counts of stomach worms, *Paramphistomum* spp., and oocysts of *Eimeria* spp. relative to the CL‐diet (*p* ≤ 0.01).

**TABLE 4:** Influence of lemongrass supplementation on serum liver enzymes and endo‐parasitic status of lambs^a^.

Variables	Diets^b^		SEM	*p*‐value
	CL‐diet	LS‐diet		
Lamb number (*n*)	10	10		
**Serum liver enzymatic activity (IU/l)**				
Alanine aminotransferase (ALT)	15.10 ± 1.20	13.74 ± 1.33	0.44	0.13
Aspartate aminotransferase (AST)	65.37 ± 1.39	62.89 ± 1.19	0.57	0.02
Alkaline phosphatase (ALP)	185.7 ± 1.79	181.9 ± 1.94	0.85	0.01
Gamma‐glutamyltransferase (GGT)	79.66 ± 1.07	78.33 ± 1.25	0.41	0.11
**Endo‐parasitic status (number)**				
Stomach worms (eggs/g faeces)	399 ± 4.30	389 ± 5.52	2.23	0.01
*Paramphistomum* spp. (eggs/g faeces)	185 ± 8.62	76 ± 6.00	18.24	<0.01
*Eimeria* spp. (oocytes/g faeces)	353 ± 11.44	272 ± 7.25	13.86	<0.01

*Note*: *p* ≤ 0.05 denotes statistically significant.

Abbreviations: IU/L = International unit per litre; g = Gram; SEM = Standard error of mean.

^a^ Non‐descript lamb *(O. aries*) indigenous to Bangladesh.

^b^ **Diets**: CL‐diet—basal diet (roughage and concentrates having 14.90% crude protein and 10.57‐mega joule metabolizable energy/kg dry matter) without lemongrass supplement; LS‐diet—Basal diet + 0.50 g lemongrass powder/kg metabolic body weight of a lamb containing 14.92% crude protein and 10.61‐mega joule metabolizable energy/kg dry matter.

### 3.5 Correlation Matrix

**FIGURE 2:**
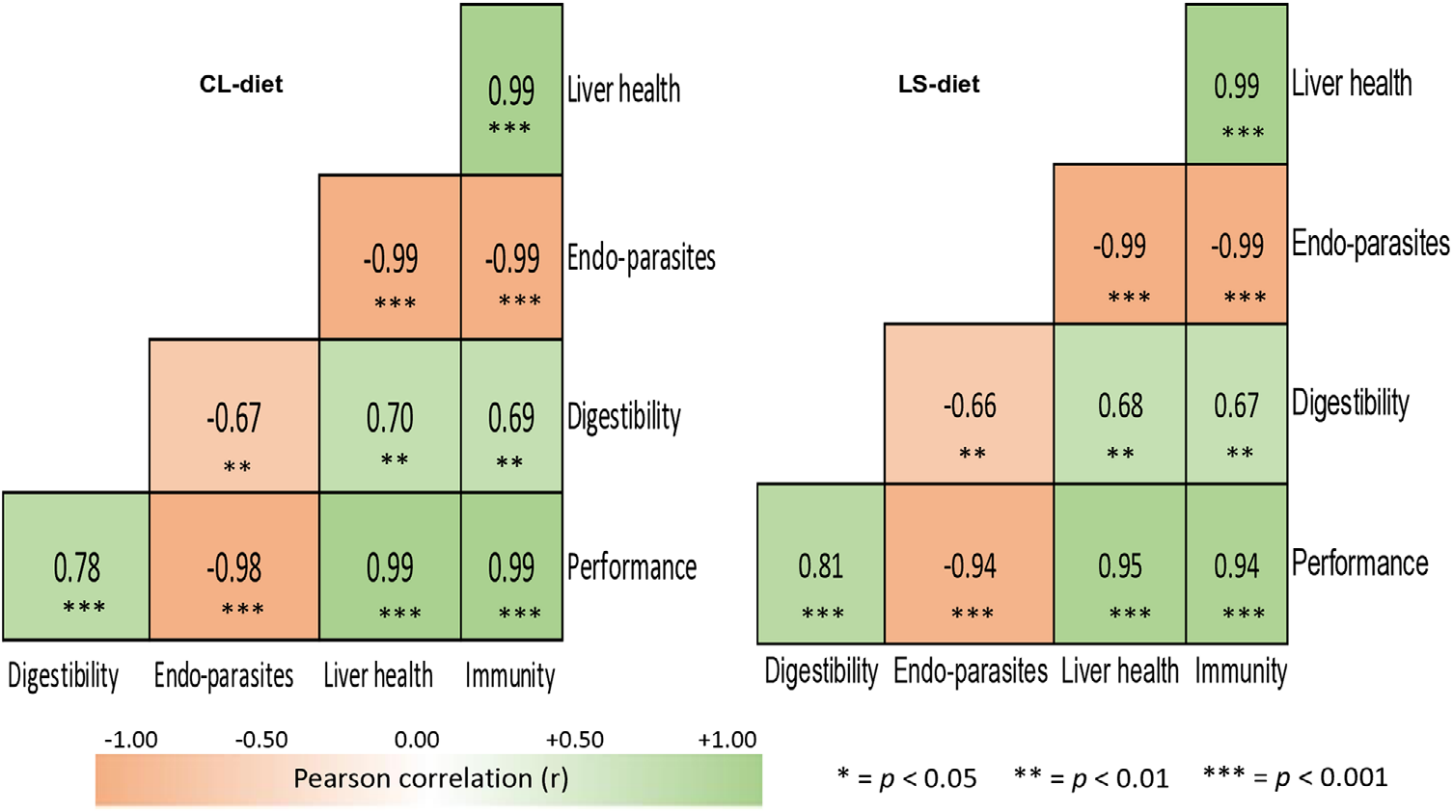
Influence of lemongrass supplementation on correlation matrix among immunity, liver health, endo‐parasites, digestibility and performance of lambs^†. †^Non‐descript lamb *(O. aries*) indigenous to Bangladesh. CL‐diet: Basal diet (roughage and concentrates having 14.90% crude protein and 10.57‐mega joule metabolizable energy/kg dry matter) without lemongrass supplement; LS‐diet: Basal diet + 0.50 g lemongrass powder/kg metabolic body weight of a lamb containing 14.92% crude protein and 10.61‐mega joule metabolizable energy/kg dry matter. *p* ≤ 0.05 denotes statistically significant.

Lambs receiving the CL‐ and LS‐diets revealed distinct relations among digestibility, endo‐parasites, liver health, immunity and performance (Figure 2). In the CL‐diet, performance showed significantly (*p* < 0.001) strong positive correlations with digestibility (*r* = 0.78), liver health (*r* = 0.99) and immunity (*r* = 0.99), but negative correlations with endo‐parasites (*r* = −0.98). Similarly, in the LS‐diet, performance showed a substantial (*p* < 0.001) positive correlation with digestibility (*r* = 0.81), liver health (*r* = 0.95) and immunity (*r* = 0.94), while exhibiting a negative correlation with endo‐parasites (*r* = −0.94).

## 4 Discussions

In this study, dietary supplementation with lemongrass tended to increase ADG, significantly enhanced growth velocity and demonstrated substantially better FCR in lambs, which supports the previous findings (Khattab et al. 2017; Allam and El‐Elaime 2020; Fahad and Al‐Wazeer 2021). These better FCR and improved growth performances of lambs in the LS‐diet may be attributed to several factors: (i) Enhanced immunity, as indicated by significantly higher level of IgG and enhanced level of IgM; (ii) improved liver health, reflected by lower AST and ALP levels; (iii) reduced endo‐parasites burden, including stomach worms and rumen fluke; and (iv) increased nutrient digestibility (Al‐Mamun et al. 2008; Mu et al. 2020; Rizwan et al. 2021; Reza et al. 2021). In addition, consistent with previous findings (Jaapar et al. 2023; Fekete and Kellems 2007), a strong positive correlation was observed among diets (CL‐ and LS‐diets), nutrient digestibility, immunity, liver health and overall performance, whereas endo‐parasites exhibited negative correlations with these parameters in the present study. This suggests that the LS‐diet, through the provision of phytochemicals, may have suppressed endo‐parasitic load and consequently improved digestibility, immunity, liver health and performance (Reza et al. 2021; Rahman, Redoy, Chowdhury, et al. 2024; Rahman, Redoy, Shuvo, et al. 2024). Furthermore, the greater nutrient digestibility and serum albumin concentrations may hasten the growth of lambs receiving the LS‐diet (Bobbo et al. 2017).

Furthermore, previous research findings (Kholif et al. 2017; Abdullah et al. 2020; Kholif et al. 2021) stated that supplementation of lemongrass with basal ration at 0.23–0.64 g per kg MBW of goats and lambs improves the DM, OM and CP digestibility, which is aligned with the current study. However, feeding lemongrass powder at 10 g per kg MBW of lambs daily has no impact on nutrient digestibility in finisher lambs (Bhatt et al. 2021), which contradicts the current study. This variation in nutrient digestibility may be attributed to differences in the doses of lemongrass used in the current (0.50 g/kg MBW) and previous study (10 g/kg MBW; Bhatt et al. 2021). Lambs fed a higher dose of lemongrass powder increase the neutral detergent fibre content of the diet, potentially reducing digestibility (Redoy et al. 2025). Nevertheless, the presence of bioactive phytochemicals in lemongrass is known to enhance nutrient digestibility (Rahman, Redoy, Shuvo, et al. 2024), which may offset this suppressive effect to some extent. Besides, daily feeding of lemongrass to Barki goats at 0.28 g per kg MBW substantially improves the nutrient digestibility (Khattab et al. 2017), which is consistent with the current study. This elevated nutrient digestibility in lambs might be attributed to the presence of phytochemicals in lemongrass, which increases the population of cellulolytic and amylolytic bacteria and reduces the proteolytic bacteria in the rumen (Wanapat et al. 2008). This lower proteolytic activity in the rumen reduces the fermentation of protein, which enhances the utilization of protein by reducing the loss of ammonia and improving microbial protein synthesis (Wanapat et al. 2008). The higher tannin contents of lemongrass bind with protein, which aids in protecting tannin‐binding salivary proteins from proteolysis in the rumen, thus enhancing the utilization of nutrients in the small intestine in ruminants (Aboagye and Beauchemin 2019).

Lemongrass contains phytochemicals that reduce the production of rumenic acid from linoleic and linolenic fatty acids by slowing the biohydrogenation process in the rumen, resulting in decreased triglycerides and LDL‐cholesterol and increased HDL‐cholesterol (Cabiddu et al. 2010), which supports the current study. Moreover, scientists revealed that feeding lemongrass to Awassi lambs (Al‐Janabi et al. 2023), Farafra ewes (Kholif et al. 2021) and tropical crossbred cows (Rahman, Redoy, Shuvo, et al. 2024) positively alters lipid profiles, consistent with current findings. Lemongrass possesses citral and alkaloids, which stimulate the activity of lipoprotein lipase enzyme and lecithin cholesterol acetyltransferase activity (Garba et al. 2020). This higher enzyme activity stimulates the triglycerides to break down into free fatty acids and glycerol, thus resulting in decreased triglycerides and LDL‐cholesterol levels (Adiputro et al. 2013).

Similar to previous findings (Khattab et al. 2017; Al‐Janabi et al. 2023), serum urea nitrogen and total protein concentration were unchanged in the LS‐diet compared to the CL‐diet, which supports the current study. Nonetheless, poly‐herbal additives substantially improve the serum albumin concentrations in lambs due to the presence of anti‐inflammatory properties (Bobbo et al. 2017; Dorantes‐Iturbide et al. 2022). Lemongrass, which contains limonene, demonstrates anti‐inflammatory activity by enhancing albumin synthesis and decreasing globulin production in dairy cows (Rahman, Redoy, Chowdhury, et al. 2024), supporting the present findings. In addition, supplementation of lemongrass at 0.50 g per kg MBW of lambs significantly increased serum IgG levels and showed the propensity of elevated serum IgM concentrations in the current study. This finding contrasts with the results of Hosoda et al. (2006), who reported that supplementing lemongrass at 1.26 g per kg MBW per day in steers has no effect on serum IgG and IgM levels. Feeding lemongrass to dairy cows at a rate of 1.05 g per kg MBW has been shown to significantly increase serum antioxidants, IgG and IgM levels (Rahman, Redoy, Chowdhury, et al. 2024; Rahman, Redoy, Shuvo, et al. 2024), which aligns with our findings. The variation in results may be attributed to the doses of lemongrass used (Zhao et al. 2022). Comparatively (300, 600, 1200 mg/kg BW), a lower dose of lemongrass essential oil (300 mg/kg BW) has been shown to most effectively reduce the production of pro‐inflammatory factors such as TNF‐α, IL‐1β and IL‐6 in mice, due to the presence of diverse phytochemicals like limonene, α‐pinene, β‐pinene, β‐laurene, γ‐terpinene and citral (Zhao et al. 2022). This reduction in inflammation likely contributes to the observed increase in serum IgG and IgM concentrations. In addition, citral, a key phytochemical in lemongrass, has been reported to enhance serum IgG and IgM levels by promoting β‐cell proliferation (Zeng et al. 2015). These factors together likely explain the higher serum IgG and IgM levels observed in lambs fed lemongrass.

Due to potent anti‐inflammatory properties, lambs receiving lemongrass exhibit lower liver inflammation, resulting in reduced serum AST and ALP levels in the existing study. However, feeding lemongrass to lambs in the current study showed no response on serum ALT and GGT levels, which partially supports the previous findings (Hosoda et al. 2006; Khattab et al. 2017). Like present findings, feeding lemongrass to Awassi lambs (Al‐Janabi et al. 2023) and cows (Rahman, Redoy, Chowdhury, et al. 2024) and administering citral to mice (Tang et al. 2018) and rats (Li et al. 2018) reduces serum AST and ALP concentrations, thereby improving liver health. This better liver health may be attributed to the presence of phytochemicals, namely, citral and limonene, in lemongrass. Both phytochemicals can reduce oxidative damage to the liver by enhancing the activity of cytochrome P450 enzymes, uncoupling protein‐2 and messenger ribonucleic acid expression (Tang et al. 2018; Mu et al. 2020).

Similar to lemongrass, plantain is an antioxidant‐rich herb having free radical scavenging activity (Al‐Mamun et al. 2007) that reduces the load of stomach worms, *Paramphistomum* spp., and *Eimeria* spp. in lambs (Reza et al. 2021), supporting the current findings. Lemongrass aqueous extract and essential oil nano‐emulsion have demonstrated anthelmintic activity against earthworms in an in vitro study (Sherwani et al. 2013) and intestinal nematodes, particularly *H. contortus* in lambs (Macedo et al. 2019). These beneficiaries’ results might be due to the presence of tannins and citral in lemongrass (Macedo et al. 2019; Rizwan et al. 2021). Tannins present in lemongrass may bind with external and internal proteins of endo‐parasites, reducing their activity and load in lambs (Molan et al. 2000). Furthermore, the high abundance of citral in lemongrass may disrupt the metabolism of endo‐parasites by inhibiting or disorganizing their important roles from the early stages of development. It can also cause nervous system derangement in endo‐parasites, inhibiting their motility (Oka et al. 2000; Macedo et al. 2019). In addition, daily supplementation of 1.05 g lemongrass in tropical crossbred cows resulted in significantly elevated serum concentrations of superoxide dismutase, glutathione peroxidase, catalase and total antioxidant capacity (Rahman, Redoy, Shuvo, et al. 2024), accompanied by improved immune responses (Rahman, Redoy, Chowdhury, et al. 2024). The observed reduction in parasitic infection risk in the present study may be attributed to the enhanced antioxidant defence system and strengthened immunity. Further research is a pre‐requisite to validate these findings.

## 5 Conclusions

Daily supplementation of lemongrass powder at 0.50 g per kg MBW in a lamb showed the propensity of DM consumption and substantially displayed better results on the FCR, ADG and growth velocity in lambs. Feeding lemongrass powder to lambs improved DM digestibility and positively influenced serum lipid, protein indices and IgG concentrations. However, lambs given lemongrass powder revealed no impact on serum urea nitrogen, total protein, IgM, ALT and GGT levels, but it significantly lowered serum AST and ALP, indicating better liver health. Lambs receiving lemongrass powder effectively reduced endo‐parasites including stomach worms, *Paramphistomum* spp. and *Eimeria* spp. These findings highlight lemongrass powder as a promising natural supplement to enhance performance and health as well as reduce endo‐parasitic load in subtropical lamb production.

## Author Contributions

**Md. Aliar Rahman**: conceptualization, methodology, writing – original draft, writing – review & editing, data curation, formal analysis. **Most Sabina Yasmin**: methodology, data curation, analysis. **Peter Wynn**: writing – review & editing. **Rakhi Chowdhury**: supervision, writing – review & editing. **Khan Md. Shaiful Islam**: supervision, writing – review & editing, supervision. **Mohammad Al‐Mamun**: conceptualization, investigation, supervision, funding acquisition, writing – review & editing, project administration, resources.

## Funding

We are grateful to the Bangladesh Agricultural Research Council for funding (Project ID: NATP2‐PBRG‐099).

## Ethics Statement

Lambs handling, faeces collection, and blood sampling were conducted by the rules of the Animal Welfare and Experimentation Ethics Committee (AWEEC/BAU/2023/62).

## Conflicts of Interest

The authors declare no conflicts of interest.

## Data Availability

All relevant data are included within the manuscript. The raw data will be available from the corresponding author upon reasonable request.
